# Possible Increased Pathogenicity of Pandemic (H1N1) 2009 Influenza Virus upon Reassortment

**DOI:** 10.3201/eid1702.101268

**Published:** 2011-02

**Authors:** Eefje J.A. Schrauwen, Sander Herfst, Salin Chutinimitkul, Theo M. Bestebroer, Guus F. Rimmelzwaan, Albert D.M.E. Osterhaus, Thijs Kuiken, Ron A.M. Fouchier

**Affiliations:** Author affiliation: National Influenza Centre and Erasmus Medical Center Department of Virology, Rotterdam, the Netherlands

**Keywords:** Viruses, reassortment, pandemic (H1N1) 2009, influenza, H1N1, H3N2, pathogenicity, transmission, ferrets, research

## Abstract

Since emergence of the pandemic (H1N1) 2009 virus in April 2009, three influenza A viruses—seasonal (H3N2), seasonal (H1N1), and pandemic (H1N1) 2009—have circulated in humans. Genetic reassortment between these viruses could result in enhanced pathogenicity. We compared 4 reassortant viruses with favorable in vitro replication properties with the wild-type pandemic (H1N1) 2009 virus with respect to replication kinetics in vitro and pathogenicity and transmission in ferrets. Pandemic (H1N1) 2009 viruses containing basic polymerase 2 alone or in combination with acidic polymerase of seasonal (H1N1) virus were attenuated in ferrets. In contrast, pandemic (H1N1) 2009 with neuraminidase of seasonal (H3N2) virus resulted in increased virus replication and more severe pulmonary lesions. The data show that pandemic (H1N1) 2009 virus has the potential to reassort with seasonal influenza viruses, which may result in increased pathogenicity while it maintains the capacity of transmission through aerosols or respiratory droplets.

The influenza virus A (H1N1) that caused the first influenza pandemic of the 21st century, pandemic (H1N1) 2009, continues to be detected worldwide (*1**,**2*). The pandemic overall has been relatively mild; disease has ranged from subclinical infections to sporadic cases of severe pneumonia and acute respiratory distress syndrome (*3**–**8*). The virus responsible is a unique reassortant virus containing neuraminidase (NA) and matrix genes from the Eurasian swine influenza virus lineage, and the other 6 gene segments are derived from the North American triple reassortant swine influenza virus lineage (*9*). From the pandemic’s start, there have been concerns the virus may mutate or reassort with contemporary influenza viruses and give rise to more pathogenic viruses.

Cocirculation of multiple strains of influenza virus A in humans provides an opportunity for viral genetic reassortment (mixing of genes from >2 viruses) (*10*). Genetic reassortment of pandemic (H1N1) 2009 virus with seasonal influenza A (H3N2) or seasonal influenza A (H1N1) viruses might thus represent a route to enhanced pathogenicity. No reassortment events between pandemic (H1N1) 2009 and seasonal viruses have been reported in humans. However, a triple-reassortant swine influenza virus A (H1N1), distinct from pandemic (H1N1) 2009 virus and containing the hemagglutinin (HA) and NA genes of seasonal influenza virus A (H1N1), was described recently (*11*). Dual infections by seasonal influenza A (H1N1) and seasonal influenza A (H3N2) viruses have been reported (*12*), as well as mixed infections of pandemic (H1N1) 2009 and seasonal influenza A (H3N2) viruses (*13**,**14*), highlighting the potential for reassortment of currently circulating influenza viruses. Subtype H1N2 reassortant influenza viruses that contain the HA of seasonal influenza A (H1N1) and the NA of seasonal influenza A (H3N2) viruses have been isolated from humans during previous influenza seasons, confirming that such HA/NA combinations can emerge in humans (*15**,**16*).

To investigate the potential for reassortment between seasonal influenza A and pandemic (H1N1) 2009 viruses, we used an in vitro selection method using reverse genetics and serial passaging under limited dilution conditions. Pathogenicity and transmission of these viruses were tested by using a ferret model. We report here the identification of 4 reassortants with different gene constellations.

## Materials and Methods

### Cells and Viruses

MDCK cells were cultured in Eagle minimum essential medium as described (*17*). Influenza virus A/Netherlands/602/2009 was isolated from the first patient with pandemic (H1N1) 2009 virus infection in the Netherlands (*18*). Influenza virus A/Netherlands/213/2003 (seasonal influenza A [H3N2]) and influenza virus A/Netherlands/26/2007 (seasonal influenza A [H1N1]) were isolated from patients during epidemics in the Netherlands. After these viruses were passaged in MDCK cells 2×, all 8 gene segments were amplified by reverse transcription–PCR, cloned in a modified version of the bidirectional reverse genetics plasmid pHW2000 (*19**,**20*), and subsequently used to generate recombinant virus by reverse genetics as described elsewhere (*19*).

### Generation of the Reassortant Viruses

Mixtures of reassortant viruses were generated in 293T cells by using reverse genetics, by co-transfecting 8 plasmids that encode the pandemic (H1N1) 2009 virus genome together with 7 plasmids encoding the seasonal influenza A (H3N2) or seasonal influenza A (H1N1) virus genome. We omitted HA of the seasonal viruses to ensure that only reassortants containing the pandemic (H1N1) 2009 virus HA could arise, against which a large proportion of the human population is still immunologically naïve (*21*). The 293T-cell supernatants were passaged in quadruplicate under limiting dilution conditions by using 10-fold serial dilutions in MDCK cells 3× to enable selective outgrowth of viruses with high in vitro replication rates. After 3 passages, the genome composition of these viruses was determined by sequencing with conserved primers targeting noncoding regions of each gene segment. Reverse genetics was also used to produce specific reassortant viruses (pandemic [H1N1] 2009–seasonal influenza A [H1N1] basic polymerase [PB] 2, pandemic [H1N1] 2009–seasonal influenza A [H1N1] PB2 acidic polymerase [PA], pandemic [H1N1] 2009–seasonal influenza A [H3N2] NA, and pandemic [H1N1] 2009–seasonal influenza A [H3N2] NAPB1) by transfection of 293T cells and subsequent virus propagation in MDCK cells.

### In Vitro Characterization of Viruses

Multicycle replication curves were generated by injecting MDCK cells at a multiplicity of infection of 0.01 50% tissue culture infective dose (TCID_50_) per cell in 2-fold (*17*). Virus titers from samples of inoculated MDCK cells, as well as nasal and throat swabs or homogenized tissue samples from inoculated ferrets, were determined by endpoint titration in MDCK cells, as described (*22*).

### Ferret Experiments

All animal studies were approved by an independent animal ethics committee. Experiments were performed under animal BioSafety Level 3+ conditions. The ferret model to test pathogenicity and transmission of pandemic (H1N1) 2009 virus was described previously (*17**,**18*). To study pathogenicity, 5 groups of 6 influenza virus–seronegative female ferrets (*Mustella putorius furo*) were inoculated intranasally with 10^6^ TCID_50_ of wild-type pandemic (H1N1) 2009 virus, or the reassortant viruses pandemic (H1N1) 2009–seasonal influenza A (H1N1) PB2, pandemic (H1N1) 2009–seasonal influenza A (H1N1) PB2PA, pandemic (H1N1) 2009–seasonal influenza A (H3N2) NA, and pandemic (H1N1) 2009–seasonal influenza A (H3N2) NAPB1, divided between both nostrils (2 × 250 μL). In the transmission experiment, 4 female ferrets for wild-type pandemic (H1N1) 2009 virus and 2 ferrets for each reassortant virus were individually housed in transmission cages and inoculated intranasally with 10^6^ TCID_50_ of virus divided between both nostrils (2 × 250 μL). Animal daily weights were used as an indicator of disease.

### Immunohistochemistry and Histopathology

Immunohistochemical testing and pathologic examination were performed by using lungs of inoculated ferrets. For each virus, 3 ferrets were euthanized at 3 and 7 days postinoculation (dpi) by exsanguination. Necropsies and tissue sampling were performed according to standard protocol. After fixation in 10% neutral-buffered formalin and embedding in paraffin, samples were sectioned at 4 μm and stained with an immunohistochemical method by using a mouse monoclonal antibody against the nucleoprotein of influenza virus A (*23*). Influenza virus antigen expression in lung sections was scored for bronchial surface epithelium, bronchial submucosal gland epithelium, bronchiolar epithelium, alveolar type I pneumocytes, and alveolar type II pneumocytes. Scoring was categorized as 0, no positive cells; 1, few positive cells; 2, moderate number of positive cells; and 3, many positive cells. Serial lung sections were stained with hematoxylin and eosin for detection and description of pathologic changes. Samples were scored for influenza virus–associated inflammation in bronchi (bronchitis), bronchial submucosal glands (bronchoadenitis), bronchioles (bronchiolitis), and alveoli (alveolitis). Scoring of severity of inflammation was 0, no inflammation; 1, mild inflammation; 2, moderate inflammation; and 3, marked inflammation. Researchers who examined the sections had no knowledge of the identity of the ferrets.

## Results

### In Vitro Selection of Reassortants

The proportion of gene segments 1–8 (except HA) analyzed was ≈60%, 60%, 65%, 90%, 95%, 100%, and 100%, respectively. Minor virus variants were not detected. No point mutations were observed in the proportions of the genome analyzed. Upon pandemic (H1N1) 2009–seasonal influenza A (H1N1) transfection and passaging, 3 reassortants contained the PB2 gene of seasonal influenza virus A (H1N1), 2 of which had also incorporated the seasonal (H1N1) PA gene. All 4 pandemic (H1N1) 2009–seasonal influenza A (H3N2) virus reassortants had the NA gene of seasonal influenza A (H3N2), and 3 of 4 reassortants also incorporated the seasonal influenza A (H3N2) PB1 gene (Table 1).

**Table 1:** Predominant virus genome composition upon in vitro selection of mixtures of pandemic (H1N1) 2009–seasonal (H1N1) and pandemic (H1N1)–seasonal (H3N2) influenza virus reassortants*

Replicates	PB2	PB1	PA	HA	NP	NA	M	NS
Pandemic (H1N1) 2009–seasonal (H1N1) 1	sH1	pH1	sH1	pH1	pH1	pH1	pH1	pH1
Pandemic (H1N1) 2009–seasonal (H1N1) 2	sH1	pH1	sH1	pH1	pH1	pH1	pH1	pH1
Pandemic (H1N1) 2009–seasonal (H1N1) 3	sH1	pH1	pH1	pH1	pH1	pH1	pH1	pH1
Pandemic (H1N1) 2009–seasonal (H1N1) 4	pH1	pH1	pH1	pH1	pH1	pH1	pH1	pH1
Pandemic (H1N1) 2009–seasonal (H3N2) 1	pH1	sH3	pH1	pH1	pH1	sH3	pH1	pH1
Pandemic (H1N1) 2009–seasonal (H3N2) 2	pH1	pH1	pH1	pH1	pH1	sH3	pH1	pH1
Pandemic (H1N1) 2009–seasonal (H3N2) 3	pH1	sH3	pH1	pH1	pH1	sH3	pH1	pH1
Pandemic (H1N1) 2009–seasonal (H3N2) 4	pH1	sH3	pH1	pH1	pH1	sH3	pH1	pH1

*PB, basic polymerase; PA, acidic polymerase; HA, hemagglutinin; NP, nucleoprotein; NA, neuraminidase; M, matrix; NS, nonstructural; sH1, seasonal influenza A virus (H1N1); pH1, pandemic (H1N1) 2009 virus; sH3, seasonal influenza A (H3N2) virus.

### In Vitro Characterization of Pandemic (H1N1) 2009–Seasonal Influenza A (H1N1) and Pandemic (H1N1) 2009–Seasonal Influenza A (H3N2) Reassortants

**Figure 1:**
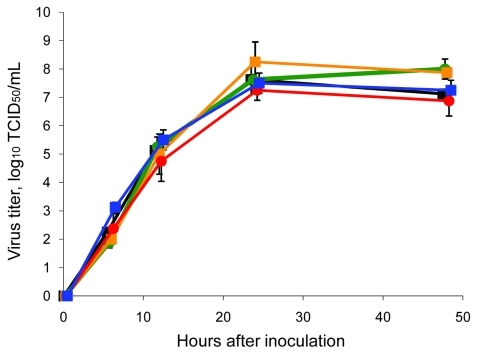
Replication of wild-type and reassortant pandemic (H1N1) 2009 viruses in MDCK cells. MDCK cells were injected in duplicate with 0.01 50% tissue culture infective dose (TCID_50_) per cell of each virus: black, wild-type pandemic (H1N1) 2009; red, reassortant pandemic (H1N1) 2009–seasonal influenza (H1N1) basic polymerase (PB) 2 acidic polymerase; blue, reassortant pandemic (H1N1) 2009–seasonal influenza (H1N1) PB2; green, reassortant pandemic (H1N1) 2009–seasonal influenza (H3N2) PB1 neuraminidase (NA); orange, reassortant pandemic (H1N1)–seasonal influenza (H3N2) NA. Supernatant samples were harvested 6, 12, 24 and 48 h after injection. Supernatant samples were titrated in MDCK cells. Geometric mean titers and standard deviation were calculated from 2 independent experiments.

The replication kinetics of pandemic (H1N1) 2009–seasonal influenza A (H1N1) PB2 and pandemic (H1N1) 2009–seasonal influenza A (H1N1) PB2PA were similar to those of wild-type pandemic (H1N1) 2009 virus, and the pandemic (H1N1) 2009–seasonal influenza A (H3N2) NA and pandemic (H1N1) 2009–seasonal influenza A (H3N2) NAPB1 reassortant viruses displayed slightly higher virus titers at 24 and/or 48 h after inoculation, with a maximum difference in virus titer of 1.0 log_10_ TCID_50_ (Figure 1). The fact that each reassortant virus replicated at least at the same rate as the wild-type pandemic (H1N1) 2009 virus agrees with results from the in vitro selection experiment described above.

### Pathogenicity of the Reassortant Viruses in Ferrets

The mean maximum weight loss was 7% for animals inoculated with the pandemic (H1N1) 2009 virus. Animals inoculated with pandemic (H1N1) 2009–seasonal influenza A (H1N1) PB2PA, pandemic (H1N1) 2009–seasonal influenza A (H1N1) PB2, pandemic (H1N1) 2009–seasonal influenza A (H3N2) NAPB1 and pandemic (H1N1) 2009–seasonal influenza A (H3N2) NA had a maximum weight loss of 4%, 2%, 2%, and 6%, respectively (data not shown).

**Figure 2:**
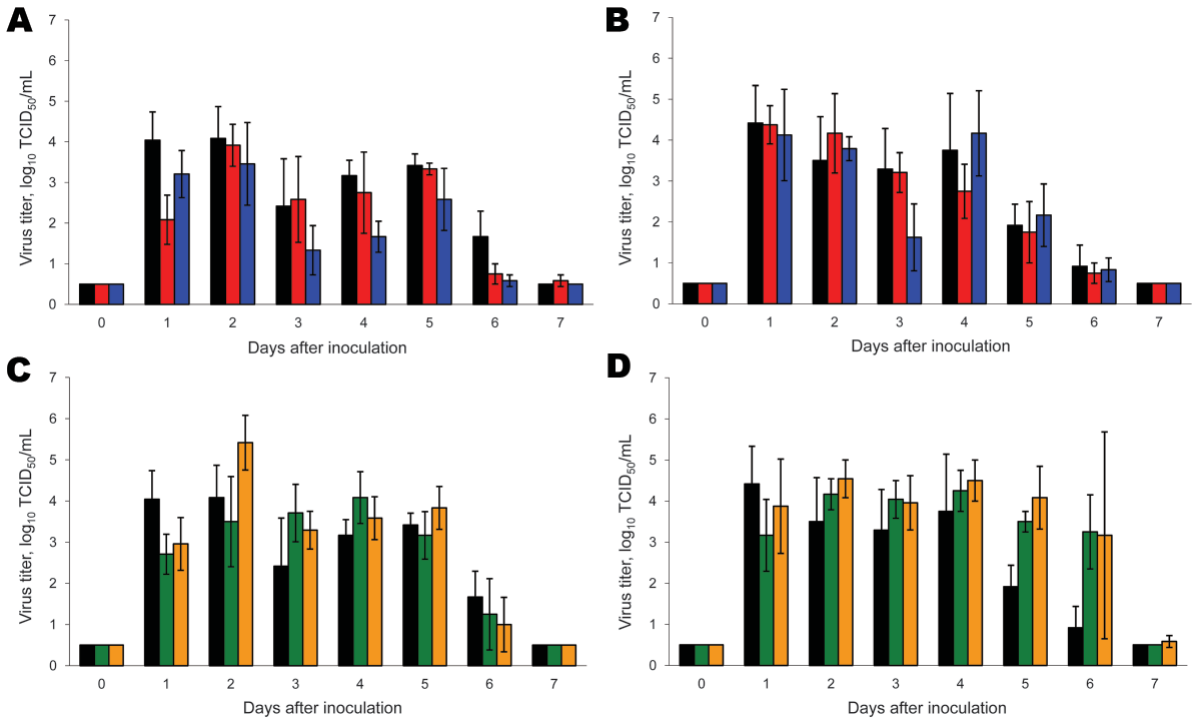
Virus shedding from the nose and throat of ferrets inoculated with wild-type and reassortant pandemic (H1N1) 2009 viruses. Virus shedding from nose (A, C) and throat (B, D) is shown for pandemic (H1N1) 2009–seasonal influenza (H1N1) (A, B) and pandemic (H1N1) 2009–seasonal influenza (H3N2) (C, D) reassortant viruses. Black, wild-type pandemic (H1N1) 2009; red, pandemic (H1N1) 2009–seasonal influenza (H1N1) basic polymerase (PB) 2 acidic polymerase; blue, pandemic (H1N1) 2009–seasonal influenza (H1N1) PB2; green, pandemic (H1N1) 2009–seasonal influenza (H3N2) PB1 neuraminidase (NA); orange, pandemic (H1N1)–seasonal influenza (H3N2) NA. Geometric mean titers are shown; error bars indicate SD. The lower limit of detection is 0.5 log_10_ 50% tissue culture infective dose/mL (TCID_50_/mL). After day 3, only 3 animals remained in each group.

Nose and throat swabs were collected daily, and virus titers were determined. Infectious virus shedding continued until 6–7 days dpi from noses (Figure 2, panels A and C) and throats (Figure 2, panels B and D) of most inoculated animals. Total virus shedding from the nose, as calculated from the area under the curve for ferrets in the experiment for 7 days (n = 3), was significantly lower in animals inoculated with the pandemic (H1N1) 2009–seasonal influenza A (H1N1) PB2 reassortant virus (p = 0.003 by *t* test) and significantly higher in the animals inoculated with the pandemic (H1N1) 2009–seasonal influenza virus A (H3N2) NA (p = 0.023 by *t* test), than in animals inoculated with wild-type pandemic (H1N1) 2009 virus. Total virus shedding from the throat, as calculated from the area under the curve for ferrets in the experiment for 7 days, was not significantly different between the groups of ferrets.

**Figure 3:**
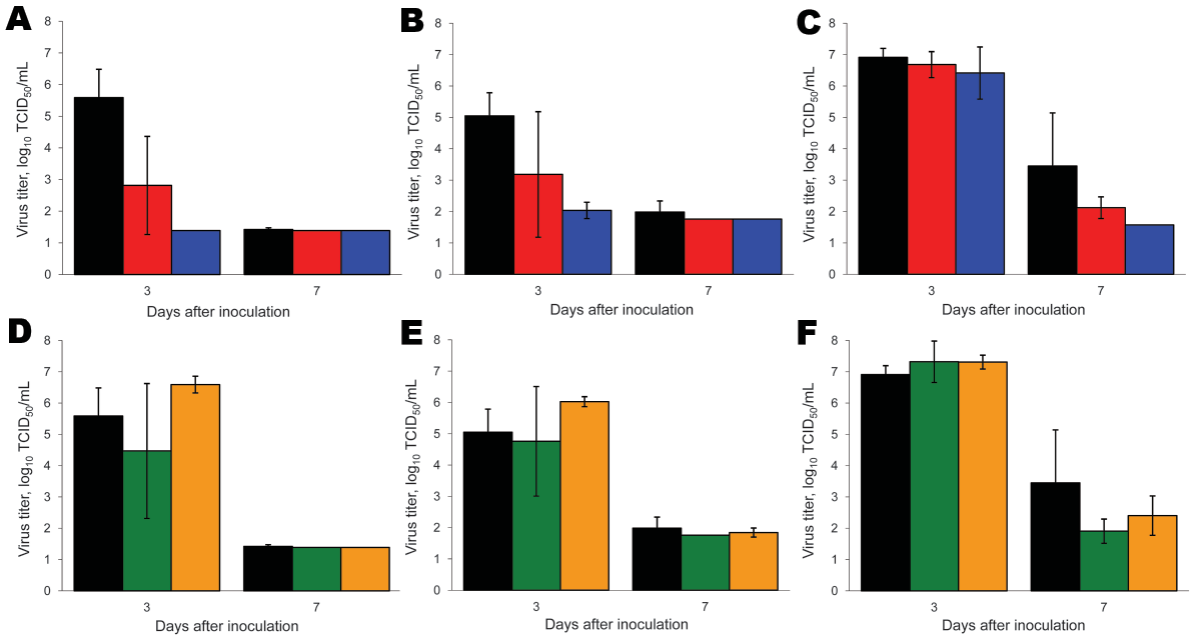
Virus detection in respiratory tissues of ferrets inoculated with wild-type and reassortant pandemic (H1N1) 2009 viruses. Virus detection in lungs (A, D), trachea (B, E), and nasal turbinates (C, F) is shown for pandemic (H1N1) 2009–seasonal influenza (H1N1) (A–C) and pandemic (H1N1) 2009–seasonal influenza (H3N2) (D–F) reassortant viruses. Black, wild-type pandemic (H1N1) 2009; red, pandemic (H1N1) 2009–seasonal influenza (H1N1) basic polymerase (PB) 2 acidic polymerase; blue, pandemic (H1N1) 2009–seasonal influenza (H1N1) PB2; green, pandemic (H1N1) 2009–seasonal influenza (H3N2) PB1 neuraminidase (NA); orange, pandemic (H1N1)–seasonal influenza (H3N2) NA. Three ferrets of each group were euthanized at 3 and 7 days postinoculation. Geometric mean titers are shown; error bars indicate SD. The lower limit of detection is 0.5 log_10_ 50% tissue culture infective dose/mL (TCID_50_/mL).

At 3 and 7 dpi, 3 ferrets from each group were euthanized, and samples from nasal turbinates, trachea, and lungs were collected for virologic examination. At 7 dpi, virus was undetectable or detected at only very low levels in these samples from all groups of ferrets. At 3 dpi, no virus or relatively low virus titers were detected in the lungs and trachea respectively, of ferrets inoculated with pandemic (H1N1) 2009–seasonal influenza A (H1N1) reassortant viruses (Figure 3, panels A and B), and titers in the nasal turbinates were similar to those for wild-type pandemic (H1N1) 2009 virus (Figure 3, panel C). These data indicate that both pandemic (H1N1) 2009–seasonal influenza A (H1N1) viruses were attenuated with respect to replication in the lower respiratory tract of ferrets.

At 3 dpi, virus was detected in the lungs, trachea, and nasal turbinates of ferrets inoculated with pandemic (H1N1) 2009–seasonal influenza A (H3N2) NAPB1 and pandemic (H1N1) 2009–seasonal influenza A (H3N2) NA viruses at approximately the same levels as upon inoculation with wild-type pandemic (H1N1) 2009 virus (Figure 3, panels D–F). Virus titers detected in the lungs and trachea of animals inoculated with pandemic (H1N1)–seasonal influenza virus A (H3N2) NA at 3 dpi were 1.0 log_10_ TCID_50_ higher than those in animals inoculated with wild-type pandemic (H1N1) 2009 virus (not statistically significant). These data indicate that both pandemic (H1N1) 2009–seasonal influenza A (H3N2) viruses tested were not attenuated in ferrets. If anything, shedding of pandemic (H1N1)–seasonal influenza A (H3N2) NA virus from the nose (Figure 2, panel C), lungs (Figure 3, panel D), and trachea (Figure 3, panel E) was higher than shedding of wild-type pandemic (H1N1) 2009 virus.

### Pathologic Changes in the Respiratory Tract of Ferrets Inoculated with Pandemic (H1N1) 2009 and Reassortant Viruses

**Figure 4:**
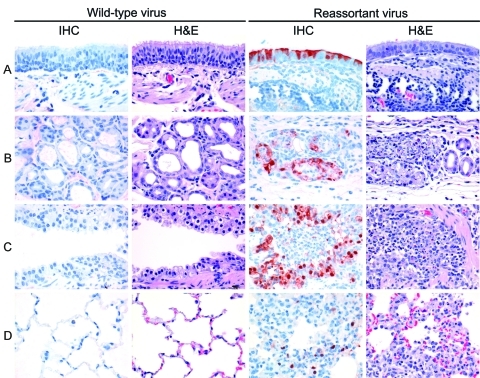
Examples of virus antigen expression and severity of lesions in different tissues of the lungs of ferrets. A) Bronchial surface; B) bronchial submucosal gland; C) bronchiole; D) alveolus. Two of 3 ferrets inoculated with wild-type pandemic (H1N1) 2009 virus had neither virus antigen expression (first column) nor associated lesions (second column) in the lung at day 3 postinoculation. In contrast, all 3 ferrets inoculated with reassortant pandemic (H1N1) 2009–seasonal influenza (H3N2) virus neuraminidase had virus antigen expression in bronchi, bronchial submucosal glands, bronchioles, and alveoli (third column), associated with epithelial degeneration and necrosis and infiltration of inflammatory cells, predominantly neutrophils (fourth column). IHC, immunohistochemistry; H&E, hematoxylin and eosin stain. Original magnification ×400.

At 7 dpi, virus antigen expression was undetectable in lung tissue of any of the euthanized ferrets, and lesions were absent or resolving. At 3 dpi, neither viral antigen expression nor lesions were detected in lungs of ferrets inoculated with pandemic (H1N1) 2009–seasonal influenza A (H1N1) PB2. Only 1 of 3 ferrets inoculated with pandemic (H1N1) 2009 had scant virus antigen expression and mild associated lesions in bronchial submucosal glands and bronchioles at 3 dpi (Table 2; Figure 4). In contrast, all 3 ferrets inoculated with pandemic (H1N1) 2009–seasonal influenza A (H3N2) NA had moderate to abundant virus antigen expression in bronchial submucosal glands, bronchioles, or both, associated with moderate to marked inflammation (Table 2; Figure 4). Virus antigen expression and associated lesions in the lungs of ferrets inoculated with pandemic (H1N1) 2009–seasonal influenza A (H3N2) PB1NA were intermediate between those of wild-type pandemic (H1N1) 2009 and pandemic (H1N1)–seasonal influenza A (H3N2) NA (Table 2).

**Table 2:** Virus antigen expression and severity of lesion in different tissues of the lung of 3 ferrets inoculated with pandemic (H1N1) 2009 or reassortant pandemic (H1N1) 2009 viruses*

Virus	Cumulative score per tissue										
	Bronchial surface epithelium			Bronchial submucosal epithelium			Bronchiolar epithelium			Alveolar epithelium	
	IHC	H&E		IHC	H&E		IHC	H&E		IHC	H&E
Pandemic (H1N1) 2009	0, 0, 0†	0, 0, 0		0, 0, 1	0, 0, 1		0, 0, 1	0, 0, 1		0, 0, 0	0, 0, 0
Pandemic (H1N1) 2009–seasonal influenza A (H1N1) PB2PA	0, 0, 0	0, 0, 0		0, 0, 0	0, 0, 0		0, 0, 0	0, 0, 0		0, 0, 0	0, 0, 0
Pandemic (H1N1) 2009–seasonal influenza A (H1N1) PB2	0, 0, 0	0, 0, 0		0, 0, 0	0, 0, 0		0, 0, 0	0, 0, 0		0, 0, 0	0, 0, 0
Pandemic (H1N1) 2009–seasonal influenza A (H3N2) PB1NA	1, 1, 0	0, 1, 0		2, 1, 0	1, 1, 0		2, 1, 0	1, 1, 0		1, 1, 0	1, 1, 1
Pandemic (H1N1) 2009–seasonal influenza A (H3N2) NA	1, 1, 1	0, 1, 1		1, 0, 3	1, 0, 3		2, 2, 3	2, 2, 3		1, 0, 1	1, 1, 1

*IHC, immunohistochemistry to detected virus antigen expression; H&E, hematoxylin and eosin staining to analyze severity of inflammation; PB, basic polymerase; PA, acidic polymerase; NA, neuraminidase. †Individual scores (as indicated in the Methods section) for 3 ferrets are listed.

Cell types in which virus antigen expression was detected were ciliated epithelial cells of bronchi, epithelial cells of bronchial submucosal glands, ciliated and nonciliated cells of bronchioles, and both squamous and cuboidal epithelial cells (interpreted as type I and type II pneumocytes, respectively) of alveoli (Figure 4). Virus antigen expression was also seen in desquamated epithelial cells and cell debris in lumina of above tissues.

Lesions associated with virus antigen expression can be categorized as acute, focal or multifocal, necrotizing bronchitis, bronchoadenitis, bronchiolitis, and alveolitis. These lesions were characterized by degeneration and necrosis of epithelial cells, infiltration of the affected tissues and their lumina by many neutrophils and few eosinophils, and exudation of edema fluid and fibrin into tissue lumina.

### Transmission of Reassortant Viruses in Ferrets

Transmission of pandemic (H1N1) 2009 and reassortant influenza viruses through aerosol or respiratory droplets was tested in the ferret model. Ferrets in groups of 4 for pandemic (H1N1) 2009 virus and 2 for the reassortant viruses were inoculated intranasally with 10^6^ TCID_50_ of virus. At 1 dpi, an uninfected ferret was placed in a cage adjacent to each inoculated ferret. All viruses were transmitted from the inoculated to the uninfected ferrets in 4/4 ferrets for pandemic (H1N1) 2009 virus and 2/2 ferrets for each of the reassortant viruses. The first day of virus detection in the previously uninfected animals was 2 days post exposure, similar for all viruses tested.

## Discussion

We used an in vitro selection method to identify reassortant viruses between pandemic (H1N1) 2009 virus and seasonal influenza A (H1N1) and influenza A (H3N2) viruses of interest for testing in a ferret model. Studying the effects of reassortment on changes in influenza virus phenotype is cumbersome because the number of reassortants that can be generated between 2 viruses is high; 2^8^ = 256 different viruses. After 3 passages, a limited number of specific virus populations were selected in vitro. Minor virus variants representing <20% of the virus population would remain undetected in our approach of PCR amplification and direct determination of the consensus sequence of the amplicons. However, upon repeating the procedure 4 times for both reassortment combinations, the seasonal influenza virus genes that were selected in the pandemic (H1N1) 2009 virus backbone were more or less consistent, with NA of seasonal influenza virus A (H3N2) being selected in 4/4 attempts, PB1 of seasonal influenza A (H3N2) and PB2 of seasonal influenza virus A (H1N1) in 3/4 attempts, and PA of seasonal influenza virus A (H1N1) in 2/4 attempts. Replication in MDCK cells may not be the best selection criterion for the identification of reassortants of interest to human health. Nevertheless, we chose this in vitro selection method because previous work has shown that pandemic (H1N1) 2009 outcompetes seasonal influenza A (H1N1) and seasonal influenza A (H3N2) viruses rapidly, reducing the opportunity for reassortment (*24*). This growth advantage over seasonal viruses was in agreement with the fact that selected viruses mostly contained pandemic (H1N1) 2009 genes. The use of reverse genetics enables production of all gene segments at approximately similar copy numbers on transfection, whereas upon double infection with 2 viruses, in vitro or in ovo viruses may differ in replication capacity, resulting in a bias of reassortants produced.

Notably, the polymerase gene segments of seasonal influenza A (H1N1) and seasonal influenza A (H3N2) viruses frequently substituted for the polymerase genes of the pandemic (H1N1) 2009 virus in vitro*.* In minigenome assays, the polymerase complex activity of the wild-type pandemic (H1N1) 2009 virus was relatively low, and replacement of various polymerase genes of the pandemic (H1N1) 2009 virus increased this activity. However, polymerase complexes with the highest activity in minigenome assays were not necessarily the ones detected in the reassortant viruses (data not shown). This apparent discrepancy is probably a result of the different parameters under investigation in the 2 assays, in particular, the production of mRNA vs. all viral RNAs.

Virus titers for pandemic (H1N1) 2009–seasonal influenza A (H1N1) PB2PA and pandemic (H1N1) 2009–seasonal influenza A (H1N1) PB2 in the lungs and trachea of ferrets were lower than titers in ferrets inoculated with wild-type pandemic (H1N1) 2009 virus, suggesting that both pandemic (H1N1) 2009–seasonal influenza A (H1N1) reassortant viruses were attenuated in ferrets, at least for replication in the lower respiratory tract. The reassortants between pandemic (H1N1) 2009 and seasonal influenza A (H3N2) viruses replicated at slightly higher rates than wild-type pandemic (H1N1) 2009 virus in vitro. Moreover, virus shedding of pandemic (H1N1) 2009–seasonal influenza virus A (H3N2) NA from the nose, lungs, and trachea of inoculated ferrets was slightly higher than wild-type pandemic (H1N1) 2009 virus. Although the differences in replication and shedding were small and not statistically significant because of the small numbers of animals in each group, the pandemic (H1N1) 2009–seasonal influenza A (H3N2) viruses were not attenuated in ferrets.

Inoculation of the reassortant pandemic (H1N1)–seasonal influenza A (H3N2) NA (either with or without the PB1 of seasonal influenza virus A [H3N2]) resulted in higher expression of virus antigen and more severe lesions at all levels of the lower respiratory tract compared with inoculation of wild-type pandemic (H1N1) 2009 virus (Table 2; Figure 4). In a previous study, the wild-type pandemic (H1N1) 2009 virus was detected more abundantly in the lower airways of ferrets than in the present study (*18*). We attribute this difference to the use of a virus isolate rather than a virus generated by reverse genetics and to a different batch of ferrets in the previous study. In the present study, all viruses were produced with reverse genetics and can thus be compared directly. Moreover, the reassortant pandemic (H1N1) 2009 virus with the NA of the seasonal influenza virus A (H3N2) was more pathogenic than both sources of pandemic (H1N1) 2009 virus, either the wild-type isolate or the virus derived by reverse genetics. Increased severity of lesions may be related to higher virus replication in the lung, to stronger host immune responses, or both (*25*).

We conclude that the pandemic (H1N1) 2009 virus has the potential to reassort with seasonal influenza virus A (H1N1) and influenza virus A (H3N2) and that such reassortment events could result in viruses with increased pathogenicity in ferrets. Although increased pathogenicity in ferrets cannot be extrapolated directly to increased pathogenicity in humans, ferrets are susceptible to natural infection and respiratory disease and lung pathology develop in a manner similar to that in humans infected with seasonal, avian, or pandemic influenza viruses. Thus, the ferret model is generally thought to be a good animal model for influenza in humans (*26**,**27*). Patterns of influenza virus attachment to cells of the respiratory tract are also similar in ferrets and humans (*28*), and the ferret model has further been used successfully for studies on virus transmission through respiratory droplets or aerosols (*18**,**29*).

All reassortants were transmitted between ferrets through aerosol or respiratory droplets. These results demonstrate that some reassortants between pandemic (H1N1) 2009 and seasonal influenza A (H3N2) were viable, remained transmissible, and were more pathogenic than the wild-type pandemic (H1N1) 2009 virus and emphasize the importance of monitoring reassortant viruses in surveillance programs because reassortment events may affect pathogenicity.

Although viruses with the NA gene (with or without the PB1 gene) of seasonal influenza A (H3N2) were identified here as potentially fit virus reassortants, reassortant viruses with other gene constellations may have selective advantages in humans as well. The 1968 influenza virus A (H3N2) pandemic also continued to reassort after the pandemic year, resulting in viruses during 1969–1971 with a different N2 gene than those earlier in the pandemic (*30*). Reassortants of influenza virus A (H1N2) with the HA of seasonal influenza A (H1N1) and the NA of seasonal influenza A (H3N2) viruses have been isolated from humans during previous influenza seasons, thereby confirming that reassortant influenza viruses with such an HA/NA combination can emerge in humans (*15**,**16*). Moreover, influenza (H1N2) viruses frequently have been detected in pigs around the world (*31*). Therefore, we recommend that reassortant of pandemic (H1N1) 2009 influenza viruses be monitored closely in surveillance programs, particularly when changes in pathogenicity or transmission in humans become apparent.
